# P-1012. The Incidence and Impact of Clostridioides difficile (C.diff) Infection following Chimeric Antigen Receptor T-Cell Therapy: A Propensity Matched Study

**DOI:** 10.1093/ofid/ofaf695.1209

**Published:** 2026-01-11

**Authors:** Sarah Ludvigsen, Senu Apewokin

**Affiliations:** University of Cincinnati, Cincinnati, Ohio; University of Cincinnati, Cincinnati, Ohio

## Abstract

**Background:**

*Clostridioides difficile (C.diff)* is a common cause of gastrointestinal infection and can carry significant morbidity and mortality in immunocompromised individuals. Patients who have received Chimeric Antigen Receptor T-cell (CAR-T) therapy represent an increasing proportion of immunocompromised hosts; however, the impact of *C. diff* in this population has not been well documented. This study aims to analyze the incidence and effect of *C. diff* infections on CAR-T therapy outcomes.

**Methods:**

TriNetX, a global health research network that provides access to electronic medical records (diagnoses, procedures, medications, laboratory values, genomic information) across large healthcare organizations (HCOs), was used to query patients who had received CAR-T therapy. Propensity score matching was performed and rates of hospitalization, admission to the intensive care unit (ICU), and mortality between patients who developed *C.diff* infection within one year of CAR-T therapy and those who did not were compared.

**Results:**

A total of 3,755 patients who received CAR-T therapy were identified across 103 HCOs. Among them, 390 (10.39%) patients were identified as having *C.diff* within one year of receiving CAR-T therapy. After propensity score matching, 309 *C.diff*-positive patients were compared with 309 matched *C.diff*-negative controls. The mean age of *C.diff*-positive patients was 59.2 years, and 60.6% were of the male sex. In comparison, the mean age of *C.diff*-negative patients was 60.7 years, with 62.3% of the male sex. There were no statistically significant differences between the cohorts in hospitalization rates (93.7% for *C.diff*-positive vs. 91.4% for *C.diff*-negative; p = 0.2781) or ICU admissions (25.5% vs. 23.8%, respectively; p = 0.6370). However, a significant difference in mortality was observed, with patients who developed *C. diff* within one year of CAR-T therapy having a higher risk of death (odds ratio: 1.834; p = 0.0009).

**Conclusion:**

This study demonstrates that *C.diff* following CAR-T therapy is not uncommon and has an impact on one year mortality. Further research is needed to investigate the correlation between *C.diff* infection and increased mortality following CAR-T therapy, as well as to determine independent risk factors for *C. diff* among this patient population.

**Disclosures:**

Senu Apewokin, MD, NESTLE: Honoraria

